# Why Not?

**Published:** 1866-10

**Authors:** 


### WHY NOT?

*A Book for every Woman.* The Prize Essay, to which the American Medical Association awarded the Gold Medal for 1865. By Horatio R. Storer, M. D., of Boston.

A little volume of 90 pages, bearing the above title, has been sent us by the publishers, Lee & Shepard, Boston, Mass.

The essay is intended to call attention, in words of condemnation, to the increasing evil of criminal abortion. We think the work should be in the hands of, not only every woman, but every man, whether physician or not. We have had evidences of a deplorable yielding, on the part of the intelligent grades of society, to the whisperings of their vicious inclinations, and feel gratified in being able to refer to a work on this subject.

The author says:

"An opinion has obtained credence to a certain extent, and it has been fostered by the miserable wretches, for pecuniary gain, at once pandering to the lust and fattening upon the blood of their victims, that induced abortions, are not unfrequently effected by the better class of physicians. Such representations are grossly untrue, for wherever and whenever a practitioner of any standing in the profession has been known, or believed to be guilty of producing abortion, except absolutely to save a woman's life, he has immediately and universally been cast from fellowship, in all cases losing the respect of his associates, and frequently by formal action, being expelled from all professional associations he may have held or enjoyed.

"The old hippocratic oath, to which each of his pupils was sworn by the father of medicine, pledged the physician never to be guilty of unnecessarily inducing miscarriage. That the standard, in this respect, of the profession of the present day has not deteriorated, is proved by the first of the resolutions adopted by the Convention at Louisville in 1859: 'That while physicians have long been united in condemning the procuring of abortion at every period of gestation, except as necessary for preserving the life of either mother or child, it has become the duty of this association, in view of the prevalence and increasing frequency of the crime, publicly to enter an earnest and solemn protest against such unwarrantable destruction of human life.' "

